# Are variations in heterotrophic soil respiration related to changes in substrate availability and microbial biomass carbon in the subtropical forests?

**DOI:** 10.1038/srep18370

**Published:** 2015-12-16

**Authors:** Hui Wei, Xiaomei Chen, Guoliang Xiao, Bertrand Guenet, Sara Vicca, Weijun Shen

**Affiliations:** 1 Key Laboratory of Vegetation Restoration and Management of Degraded Ecosystems, South China Botanical Garden, Chinese Academy of Sciences, Guangzhou 510650, China; 2 School of Geographical Sciences, Guangzhou University, Guangzhou 510006, China; 3 Laboratoire des Sciences du Climat et de l′Environnement, Centre National de Recherche Scientifique, Gif Sur Yvette 91190, France; 4 Research Group of Plant and Vegetation Ecology, Department of Biology, University of Antwerp, Universiteitsplein 1, 2610 Wilrijk, Belgium; 5 University of Chinese Academy of Sciences, Beijing 100049, China

## Abstract

Soil temperature and moisture are widely-recognized controlling factors on heterotrophic soil respiration (R_h_), although they often explain only a portion of R_h_ variability. How other soil physicochemical and microbial properties may contribute to R_h_ variability has been less studied. We conducted field measurements on R_h_ half-monthly and associated soil properties monthly for two years in four subtropical forests of southern China to assess influences of carbon availability and microbial properties on R_h_. R_h_ in coniferous forest was significantly lower than that in the other three broadleaf species-dominated forests and exhibited obvious seasonal variations in the four forests (*P* < 0.05). Temperature was the primary factor influencing the seasonal variability of R_h_ while moisture was not in these humid subtropical forests. The quantity and decomposability of dissolved organic carbon (DOC) were significantly important to R_h_ variations, but the effect of DOC content on R_h_ was confounded with temperature, as revealed by partial mantel test. Microbial biomass carbon (MBC) was significantly related to R_h_ variations across forests during the warm season (*P* = 0.043). Our results suggest that DOC and MBC may be important when predicting R_h_ under some conditions, and highlight the complexity by mutual effects of them with environmental factors on R_h_ variations.

With about 68–79 petagram (Pg) CO_2_-C being released into the atmosphere each year, soil respiration is the second largest carbon (C) flux in the terrestrial C cycling^1^,^2^. Because variations in soil respiration are most likely to upset the equilibrium of terrestrial C cycling^3^,^4^, soil respiration has received increasing concerns in recent decades^5^. Total soil respiration (R_s_) consists of two components, i.e., autotrophic (R_a_) and heterotrophic (R_h_) respiration, with R_a_ mainly being the CO_2_ released by plant roots and R_h_ by microbial activities in association with soil organic matter (SOM) decomposition^6^,^7^. Averaged on the global scale, R_h_ accounts for 54% of R_s_ in forests^6^. Temperature and water supply, interactively or separately, have been most frequently reported to control soil respiration^1^,^8^,^9^, and account for a certain portion of variations in soil respiration in previous studies^5^,^8^,^10^. However, which factors contribute to the remaining proportion is still an active topic.

Substrate availability is proposed as an important underlying factor influencing soil respiration^5^,^11^, and even overshadows the role of temperature in the processes of soil respiration in specific ecosystems^12^,^13^. A positive substrate-R_h_ relationship has been found under laboratory conditions, especially when soil microbes are energy-limited^14^. The increase in CO_2_ efflux rate with C additions may be attributed to reactivations of dormant microorganisms^15^, augments in microbial biomass and activity^14^,^16^, and shifts in microbial community composition and growth strategies^17^. Substrate-dependent soil respiration was also noted in field studies^12^,^18^, but this was often based on statistically significant relationships between soil respiration rate and primary productivity^12^, photosynthates^18^, or litter production^19^. Caution should be taken when one would extend to a conclusion that substrate availability significantly influences R_h_, because soil substrate content is not always significantly related to vegetation productivity^20^. Few studies have related periodically-measured R_h_ to the according soil substrate content in fields, as did in the study of Iqbal *et al*.^21^.

In natural ecosystems, soil organic C constitutes of various C-contained compounds. In spite of being analysed by means of diverse extraction/assay procedures in different studies, SOC can be pooled as labile and recalcitrant SOC, or in some cases named as fast and slow turnover C pools etc.^22^. Labile (or fast turnover) SOC fractions can be easily utilized while recalcitrant (or slow turnover) C compounds are often hardly-decomposed by soil microorganisms^17^. Consequently, differences in the content of these SOC fractions may result in differed soil respiration, especially heterotrophic respiration, due to the nature of decomposability and preference by soil microorganisms. Labile SOC content, e.g., represented by the concentration of dissolved organic C or KMnO_4_-oxidized OC, was observed to related significantly to microbial respiration rate across sites or soil depths^11^,^23^. The amount of labile C inputs to soils may affect organic C decomposition in various ways, e.g., low concentrations labile C input stimulating decomposition while high concentrations C input inhibiting decomposition^17^. However, Cheng *et al*.^24^ found that available C did not limit R_h_ in the rhizosphere. To clarify the substrate-soil respiration relation, further studies remain needed, especially in natural ecosystems where the input of organic materials (e.g., litter and root exudates) may greatly affect the forms and amounts of substrate availability to respiration.

Traditionally, the first order decay functions that take into account of the temperature and moisture effects are often used in modelling soil respiration^25^,^26^. Soil microbial properties including microbial biomass, community composition, and enzyme kinetics are considered important for predicting R_h_ and SOC storage in recent studies^25^,^27^,^28^, but they are still poorly represented in terrestrial C cycling models^29^. Comparing with microbial community composition and enzyme kinetics which are complex to assay and hard to parameterize for models, microbial biomass has been firstly targeted in most of microbial-based models^29^. This is attributable to that, on the one hand, soil microbial biomass C *per se* is an important active SOC pool^30^ and on the other hand, it is considered as an important agent of SOC sequestration since increasing evidences show that microbial-derived OC contributed greatly to the stable SOC pool^31^. However, Luo *et al*. argued that the first order decay functions should be kept as the backbone of terrestrial C models, since empirical evidences for those complex microbial processes remain rarely observed in natural ecosystems^26^. Birge *et al*. also suggested to omit microbial biomass from biogeochemical models, based on the results of an incubation experiment^32^. Whether microbial properties such as microbial biomass dynamics should be incorporated into models remain under debate.

Here, we conducted a field investigation on soil respiration twice per month and other soil physiochemical properties once a month for two years in four subtropical forests of southern China. This region is a significant C sink^33^ and therefore potentially important to global C-budget. The studied forests including a pristine broadleaved forest (BF), a secondary mixed coniferous and broadleaved forest (CF), a plantation with coniferous tree species (CP), and a plantation with broadleaved tree species (BP) are representatives of major forest types in the lower subtropical China^34^,^35^. This study was conducted with three objectives: the first was to observe seasonal and forest-type R_h_ variability in the four subtropical forests, the second to explore potential relationships between substrate availability and the R_h_ variations, and the third to clarify the role of microbial biomass in soil respiration processes. We aimed to provide empirical evidences for whether substrate availability and microbial biomass C should be taken into account in terrestrial C cycling models.

## Results

### Temporal variations in R_h_ and environmental conditions

**Figure 1:**
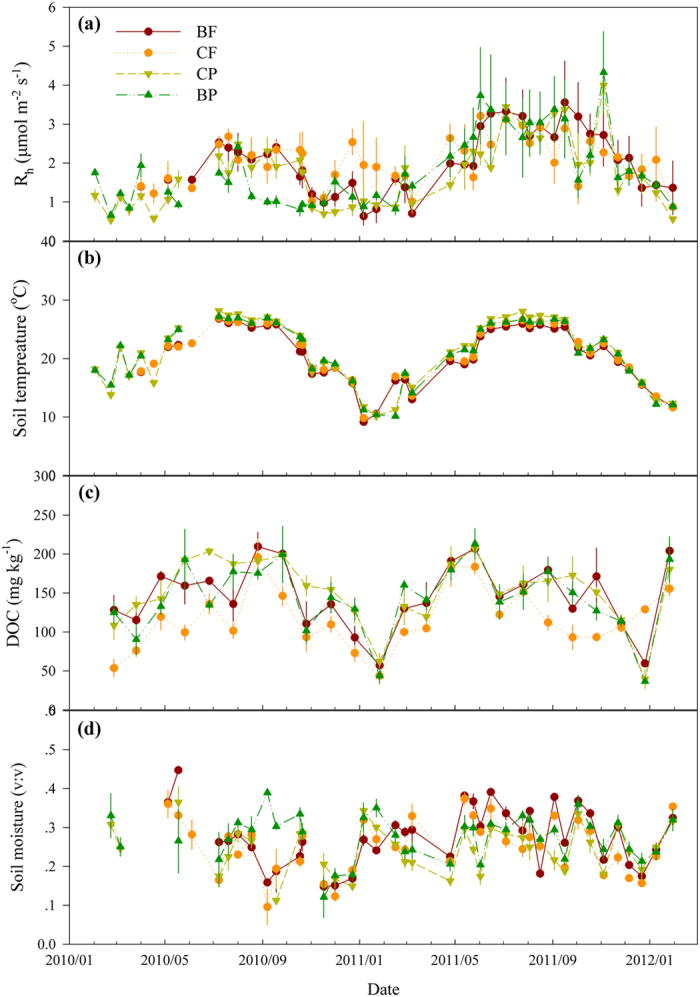
Heterotrophic soil respiration rate (R_h_, a), soil temperature (b), dissolved organic carbon (DOC, c), and soil moisture (d) recorded in the four forests over time. Data points are means and error bars represent standard errors (*n* = 3). Measure period ranges from February 2010 through January 2012. The BF is the monsoon evergreen broadleaved forest, CF the mixed coniferous and broadleaved forest, CP the plantation with mixed conifers, and BP the plantation with mixed native broadleaved species. The abbreviations for the four forests (BF, CF, CP, and BP) are the same in the following figures.

Over the two years, air temperature (T_air_) recorded in a neighbor weather station exhibited an obvious unimodal seasonal fluctuation in each year, with the average T_air_ being 27.0 °C in the warm-wet season (April to September, termed as warm season hereafter) and 17.3 °C in the cool-dry season (the other months in a year, as cool season hereafter) (Supplementary Fig. S1). Surface soil temperature at 5 cm depth (T_5_) observed at our study sites showed a similar temporal pattern and was strongly correlated with T_air_ (*P* < 0.001, Supplementary Figs S1 and S2). In all the forests, T_5_ in the cool season was significantly lower than that in the warm season (*P* < 0.001). Although most of the precipitation occurred in the warm season at the four sites (Supplementary Fig. S1), soil moistures at 5 cm depth (M_5_) were significantly different between the warm and cool seasons in the BF (*P* = 0.045) and not in the other three forests (*P* = 0.101 for CF, *P* = 0.844 for CP, and *P* = 0.159 for BP; Fig. 1d).

Heterotrophic soil respiration exhibited significant seasonal patterns all the four forests (*P* < 0.001, Fig. 1a and Table 1), which coincided well with T_5_ (comparing Fig. 1a with 1b). Consistent with R_h_, DOC content showed obvious seasonal patterns over the investigation period (*P* < 0.001, Fig. 1c and Table 1), with higher content in the warm than in the cool season (*P* < 0.05). Other substrate indices including readily-oxidizable organic C (ROC), non-readily oxidizable organic C (NORC), total organic C (TOC), and total nitrogen (TN) changed significantly over time (*P* ≤ 0.001, Table 1 and Supplementary Fig. S3), but the magnitude of variations was not as high as that in R_h_, T_5_, and DOC (comparing Supplementary Fig. S3 with Fig. 1). In contrast, soil MBC exhibited negligible seasonal fluctuations in the studied forests (*P* = 0.204, Table 1 and Supplementary Fig. S3).

### Forest-type variations in R_h_ and edaphic properties

**Figure 2:**
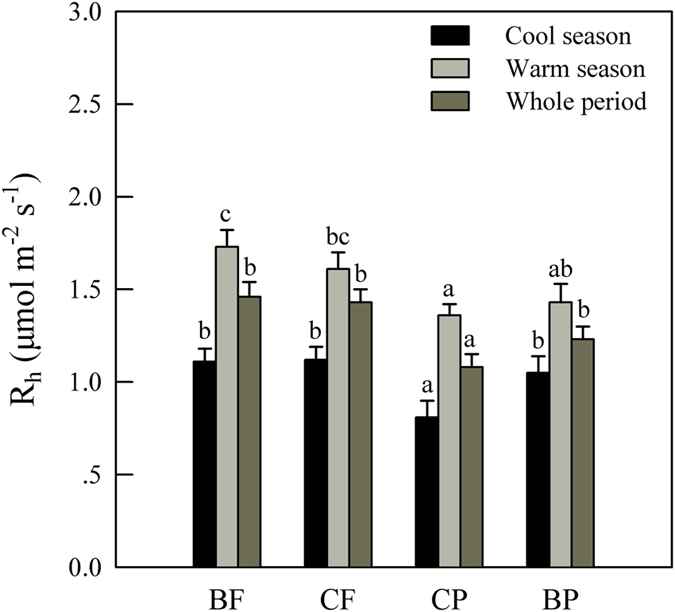
Average heterotrophic soil respiration rate (R_h_) at seasonal and measurement-period scales in the four forests. Vertical columns are means and error bars represent standard errors. Different letters above bars indicate significant differences at *P* < 0.05 among forests on the same time scale. The warm season ranges from April to September, while the other months in a calendar year compose the cool season.

Average R_h_ was 1.40, 1.36, 1.08, and 1.23 μmol m^−2^ s^−1^ for the BF, CF, CP, and BP, respectively (Fig. 2). Respiration rate was significantly lower in the CP than in the BF, CF and BP (*P* < 0.05), but significant difference was not observed in R_h_ among the latter three (*P* > 0.05, Table 1 and Fig. 2). An exception was in the warm season when significantly higher R_h_ was observed in the BF than in the BP (Fig. 2). Approximately, annual CO_2_-C released via microbial respiration was 5.2 megagram (Mg) C per hectare per year in these forests (Supplementary Table S2).

**Figure 3:**
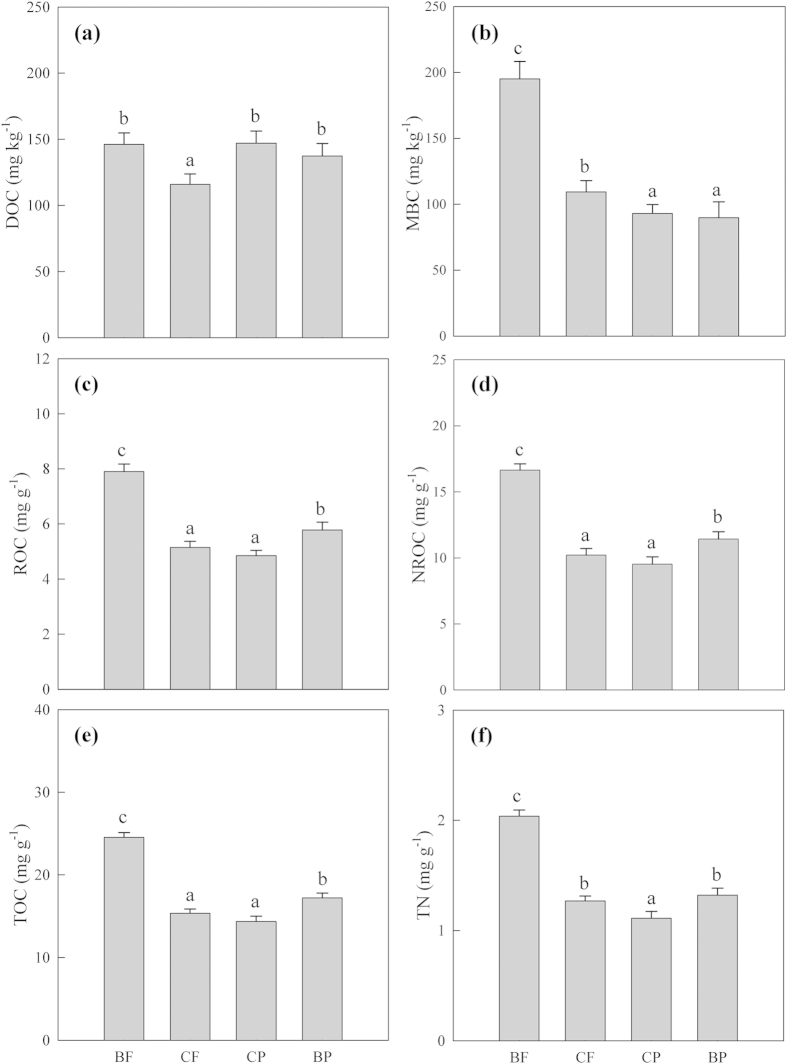
Average soil carbon (C) and nitrogen (N) content and microbial biomass C (MBC) in the four forests. Vertical bars are means within the 2-year measurement period and error bars represent standard errors (*n* = 24). Different lowercase letters above bars indicate significant differences at *P* < 0.05 in each panel. DOC is dissolved organic C, ROC readily-oxidizable organic C, NROC non-readily oxidizable organic C, TOC total organic C, and TN total nitrogen.

Soil temperature was significantly higher in the two plantations (i.e., CP and BP) than in the secondary mixed and primary forests (i.e., CF and BF; *P* < 0.001), whereas moisture was not significantly different among forests (*P* = 0.708, Table 1). Soil substrate content and MBC differed significantly among the forests (*P* < 0.05, Table 1 and Fig. 3). On average, the BF had the highest content of ROC, NROC, TOC, and TN, followed by the BP, CP, and CF orderly (*P* < 0.05, Fig. 3). Soil MBC was also the highest in the BF but lowest in the two plantation forests (*P* < 0.05, Fig. 3). Soil DOC concentration was significantly lower in the CF than in the other three (*P* < 0.05), but did not significantly differ among the BF, CP, and BP (*P* > 0.05, Fig. 3).

### Underlying factors relating to the R_h_ variations

**Figure 4:**
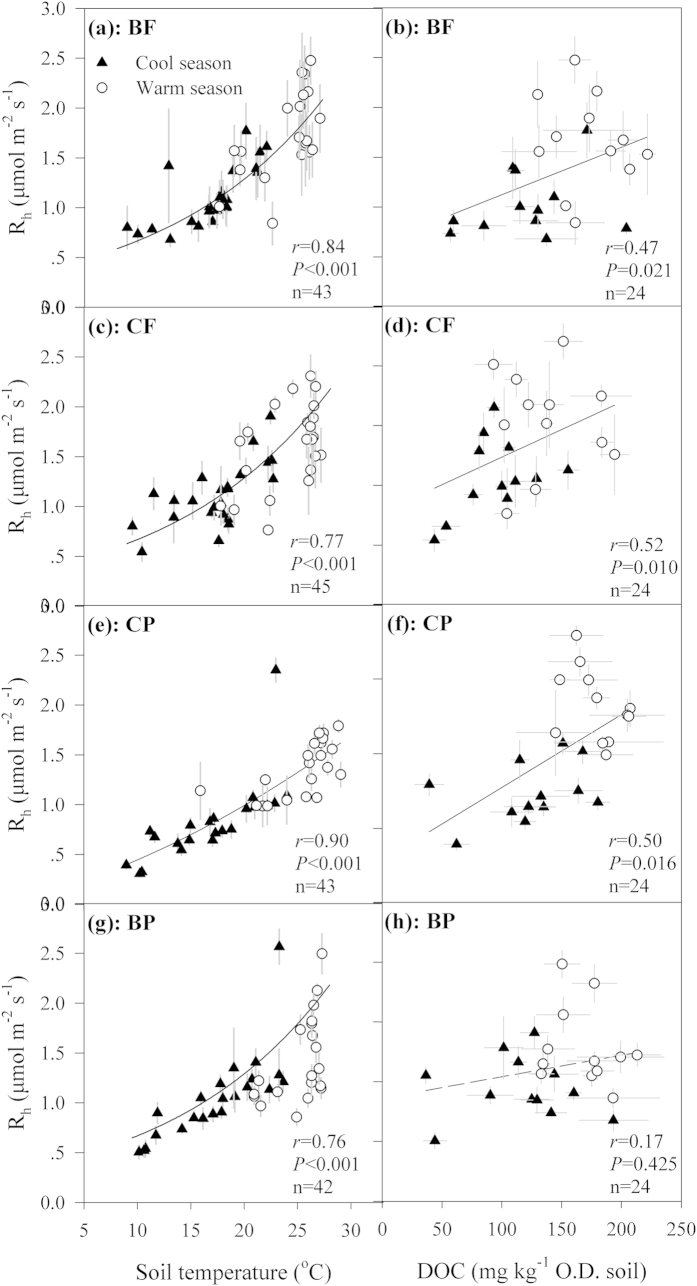
Relationships between soil temperature or dissolved organic carbon (DOC) and heterotrophic soil respiration (R_h_) in the four forests. Plots are means and error bars represent standard errors (*n* = 3). Solid lines indicate significant relationships between the two variables. Statistical Pearson coefficients *r*, *P* value and number of samples are shown in each panel. In prior to correlation between temperature and R_h_, R_h_ was ln-transformed due to the obvious exponential relationship between both. O.D. soil in the unit of DOC is the abbreviation of oven-dried soil.

In the four forests studied, soil temperature was the dominant influential factor controlling the seasonal R_h_ variations, of which 45–85% was explained by T_5_ changes with exponential functions (*P* < 0.001, Fig. 4). Soil moisture did not show a significant relationship with R_h_ in any of the forests within our investigation period (*P* > 0.05). Besides soil temperature, DOC content was also significantly related to the seasonal R_h_ variations in these forests (*P* < 0.05) except for the BP (*P* = 0.425, Fig. 4), but such significant relationships did not maintain (*P* > 0.05) after removing the temperature effect on R_h_ firstly employing statistical techniques (i.e., partial mantel test and partial correlation analysis). No significant correlations was detected between R_h_ and the other substrate indices in the cool and warm seasons, or the whole period (*P* > 0.05, Supplementary Fig. S4). Neither did microbial biomass C show a significant correlation with the seasonal R_h_ variations in any of the studied forests (*P* > 0.05, Supplementary Fig. S4).

**Figure 5:**
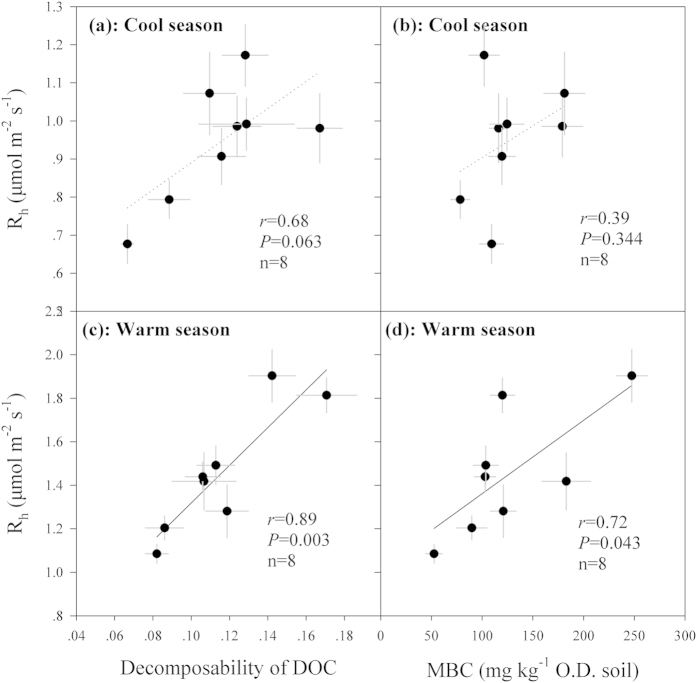
Correlations between heterotrophic soil respiration (R_h_) and decomposability of dissolved organic carbon (DOC) or microbial biomass carbon (MBC) across the four forests in the cool or warm season, respectively. Plots represent means in the cool or warm season in each year and error bars represent standard errors (n = 6). Solid lines indicate significant relationships at *P* < 0.05 while dashed lines indicate insignificant relationships. Statistical Pearson coefficients *r*, *P* value and number of samples are shown in each panel.

Across the forests, no significant correlations were observed between R_h_ and substrate content or C/N ratio, either in the cool or warm season (*P* > 0.05, Supplementary Fig. S5). Microbial biomass C was significantly related to R_h_ in the warm season (*P* = 0.043, Fig. 5). At presence, specific respiration rates and microbial respiration quotient were calculated to indicate decomposability of OC and microbial metabolic activity^23^,^36^,^37^. Results showed that decomposability of DOC was significantly related to R_h_ across the forests in the warm season (*P* = 0.003), and marginally significant in the cool season (*P* = 0.063, Fig. 5). However, no significant relationships were detected between R_h_ and decomposability of ROC and TOC (*P* > 0.05). Microbial respiration quotient did not show significant correlation with R_h_ in either of the seasons (*P* > 0.05).

## Discussion

In these subtropical forests, CO_2_ originated from microbial decomposition on average accounted for 60% of the total soil respiration, which was approximately 8.6 Mg C ha^−1^ yr^−1^ (Wei *et al*., unpublished data). The annual R_h_ and its percentage to R_s_ observed in the present study are in the range reported by previous studies^5^,^38^ but a bit higher than the corresponding averages of global subtropical ecosystems (4.1 Mg C ha^−1^ yr^−1^ and 53%, respectively)^5^. However, subtropics constitute of humid and semiarid/arid ecosystems, with the former predominated by evergreen broadleaved forests while the latter by savannas or deserts^39^. Therein, soil respiration in humid broadleaved forests is significantly higher than that in arid ecosystems such as savanna^40^ and the relatively higher R_h_ in this study may be attributable to sufficient supply of water and heat in these forests^34^, which is beneficial for microbial activities and SOM decomposition. Moreover, different vegetation productivity in these subtropical ecosystems^39^,^40^, consequently different qualities and quantities of organic C substrates input into soil, could also contribute to the discrepancy between this study and the global average R_h_.

One aim of this study was to detect R_h_ variability on the temporal and spatial scales in the four subtropical forests. As shown, heterotrophic soil respiration exhibited obvious seasonal rhythms in all of the forests studied (Fig. 1a). Our results indicated that soil temperature was the predominant influential factor driving the seasonal R_h_ variations. This exponential relationship between both is well consistent with previous soil respiration studies^10^,^41^,^42^. Theoretically, temperature rises within a certain range can stimulate metabolism activities and maintenance respiration of soil microorganisms^43^; both processes would result in elevated R_h_.

Soil water supply can influence soil respiration in many ecosystems^8^,^9^,^44^, but relationships between the two are diverse^9^,^44^,^45^. For example, linear, exponential, and quadratic functions have been used to fit the relationships between soil respiration and moisture previously^9^,^45^. However, we did not observe an obvious moisture effect on R_h_. That may be attributed to the relatively high soil moisture recorded within our investigation period (averagely 27%, 29%, 29%, and 28% in the BF, CF, CP, and BP, respectively). Even in the cool season with less precipitation, average M_5_ was 26%, 29%, 27%, and 27% for the four forests, respectively. Soil respiration variations may be more determined by soil moisture as far as it exceeds a specific lower (e.g., 15%)^46^ or upper threshold^8^, which depends greatly on soil properties^44^. Soil moisture at our sampling points may have rarely reached the threshold to limit the activity of soil microorganisms, just as in some previous studies^8^,^47^,^48^. Therefore, the relatively high M_5_ may explain the absence of moisture effect on soil respiration observed in this study.

Another aim of our study was to verify the relationship between substrate availability to the R_h_ variability under field conditions. To date few studies have related periodically-measured R_h_ to the corresponding soil substrate (but see Iqbal *et al*.^21^), despite that substrate supply has been considered a marked influential factor on soil respiration^12^,^19^,^49^. Therefore, we assayed different forms of SOC together with R_h_ in these subtropical forests to reveal direct linkages between the two. In this study, DOC was chosen as an index of soil labile C supply^23^,^50^,^51^, since it could support a great proportion of microbial activity in soils^52^,^53^, especially in the initial stage after it enters the soil^50^, and respiration CO_2_-C was observed to originate from DOC^54^. We recognized that part of DOC in mineral soils could originate from decomposition of litter and SOM^53^, implying that DOC, at least in part, was likely products of SOM decomposition, but other processes such as leaching of soluble organic materials from litter, root exudations, and microbial cell lysates could also contribute to DOC pool in soils^52^,^53^. As results showed, the positive correlation between DOC content and R_h_ indicates that the amount of soil substrate supply may positively affect microbial respiration, with higher DOC content stimulating microbial respiration. Notably, however, this significant relationship between R_h_ and DOC by simple correlation analysis did not maintain after removing the compound effect of temperature on R_h_ employing statistically partial mantel test or partial correlation analysis, taking into account that soil temperature also fluctuated consistently with DOC content within the period. This would not completely deny the underlying effect of DOC content on R_h_ but highlights the confounding effects of multi-variables on soil respiration in field, and therefore the pattern needs to be interpreted with care. How to efficiently differentiate the compound effects of targeted and synergetic variables has been a challenge in field studies.

Dissolved OC quality, indicated by decomposability of DOC^23^,^36^,^37^, was found to affect R_h_ significantly across forests. Soil microorganisms may incorporate high-quality DOC efficiently into their biomass; this means that with the equal amount of DOC supply, less C would be emitted as CO_2_ in high vs. low quality DOC. However, the significant correlation between them was observed only in the warm but not the cool season, possibly due to some other variables interactively affecting R_h_ with the quality of DOC in the cool season and therefore shadowing its role then. Soil ROC was another index employed to indicate substrate availability, as it is highly labile for decomposition and sensitive to reveal changes in pool size^55^. Nevertheless, we did not observe a significant correlation between ROC and R_h_ in any forest. Soil DOC is a preferable predicting variable for microbial respiration over ROC, NROC, and TOC, possibly due to it being highly movable and thus easily-available to soil microorganisms^54^. These observations prove that soil substrate availability contributes to the R_h_ variations to a certain extent in forests. However, we cannot rule out the possibility that the good correlation between DOC content and R_h_ is attributable to that both of DOC and CO_2_ are products of SOM decomposition in this study. Further studies using isotope tracing technique to clarify the relationship between DOC and respired CO_2_-C could improve our understanding on this topic.

Although both soil temperature and DOC content showed positive correlations with R_h_ in these forests, neither of them could explain the R_h_ variations across the four forests. Instead, soil TOC and its readily-oxidizable fraction, as well as TN and microbial properties, may be associated with the R_h_ differences among the four forests. For example, the highest R_h_ in the BF corresponded with the highest substrate supply, since the concentrations of soil TOC, ROC, and TN were significantly higher in the BF than in the other forests. Higher R_h_ in the BF may also be ascribed to its higher MBC compared with the other forests, because MBC was significantly related to R_h_ (Fig. 5d; see also Wang *et al*.^11^). Similarly, the R_h_ differences between CP and CF or BP are likely attributed to the lower MBC or substrate supply in the CP. Moreover, we consider that OC decomposability may also contribute to the observed R_h_ variations among forests. Soil microbes with low C use efficiency (CUE) may exhibit higher R_h_ even when less substrate is supplied^56^,^57^, because they incorporate SOC into their biomass with low-efficiency. We did not measure CUE directly but the decomposability of OC may indicate microbial CUE indirectly, with higher R_h_/OC implying lower CUE. As expected, the CF had the highest decomposability of OC, implying that soil microbes in CF had the minimum efficiency in incorporating OC into their biomass. In other words, they contain higher efficiency in converting OC into CO_2_, which may explain why the CF with lower substrate supply had higher R_h_ than that in BP. Assays in microbial community composition with C use efficiency/preference would provide stronger evidences for this speculation but unfortunately, they were not covered in the present study.

Moreover, our results suggest that incorporating MBC into terrestrial C cycling models may improve the prediction accuracy for R_h_, but this would not be always the case since we observed a significant correlation between MBC and R_h_ only in the warm season. This partially supports to take MBC into account when modeling soil respiration^29^,^30^. However, in specific ecosystems or under specific environmental conditions, MBC could only be a minor factor controlling R_h_ when some other variables influence R_h_ greater over MBC^32^. Empirical evidences remain insufficient to verify underlying importance of microbial properties on soil respiration in natural ecosystems^26^, although the results of this study increase the profile for supporting to incorporate MBC in terrestrial C cycling models. Additionally, the observations in the present study that significant correlations between R_h_ and MBC or the decomposability of DOC occurred in the warm season but not in the cool season highlight the complexity to explore influential factors controlling soil respiration in field, as differed variables may play roles on affecting R_h_ within different periods even in one ecosystem^45^, and this is true in different ecosystems^17^,^18^,^24^. There remains a challenge to establish a versatile model for predicting soil respiration accurately under different conditions in various ecosystems, because it is always hard for the selection of optimized parameters to balance the accuracy and utility of models^30^.

In summary, heterotrophic soil respiration showed obvious temporal and forest-type variations in these subtropical forests within our investigation period. Temperature was the dominant factor controlling R_h_ variations in these forests. Soil moisture did not affect R_h_ within our investigation period since the water supply rarely went beyond or below the thresholds to limit microbial activities. In spite of the confounding effects with temperature, soil substrate availability may be also an important influential factor controlling soil respiration, as indicated by the synchronical seasonal changes in DOC content and R_h_ and the preferable correlation between R_h_ and DOC over ROC, NROC, or TOC. Moreover, incorporating MBC into C cycling models could improve the prediction of R_h_ variations but it depends case by case. Further empirical studies combining incubation experiments and field observations remain needed to explore how substrate availability and microbial properties affect R_h_.

## Methods

### Site description

Field investigations were conducted in four subtropical forests in Guangdong province, southern China. The BF and CF forests are situated in the Dinghushan Biosphere Reserve (DBR; 112°30′ ~ 112°33′E, 23°09′ ~ 23°11′N), and the CP and BP plantations are located at the Heshan National Field Research Station of Forest Ecosystems (HSF, 112°54′E, 22°41′N), which is about 70 km away from the DBR. Both sites experience subtropical monsoon climate, with average air temperatures of 22.3 °C in the DBR and 21.7 °C in the HSF^58^,^59^. Average annual precipitation amount is 1678 mm in the DBR and around 1700 mm in the HSF, of which about 80% falls in the warm season ranging from April to September^58^,^59^. The remaining 20% precipitates in the other months in a calendar year which compose the cool season. Soil in both sites is acidic and classified as oxisols in the USDA soil taxonomy^34^,^35^.

The BF is the regional climax vegetation in lower subtropical China. Due to the protection by Buddhist monks in a nearby temple, the BF under study has developed naturally for about 400 years^34^. The CF has developed gradually from a planted coniferous forest with the invasions of pioneer broadleaved species since it was free from human disturbances 70 years ago^34^. The stand age of CF is around 100 years old. The dominant tree species are *Castanopsis chinensis*, *Schima superba*, *Cryptocarya chinensis*, *Machilus chinensis*, and *Syzygium rehderianum* in the BF and *Pinus massioniana* and *S. superba* in the CF^10^,^19^,^34^.

Before plantation, the HSF site was a homogenous degraded hilly land with similar soil property and species composition. The BP (2.68 ha) and CP (3.17 ha) were established in 1984 to experimentally demonstrate forest restoration in such degraded hilly land in southern China. In each of the plantations, trees were planted at a spacing of 3 × 2.5 m, with the dominant species being *P. massoniana* and *Cunninghamia lanceolata* in the CP, and *S. superba* and *Cinnamomum burmanii* in the BP^59^,^60^. The species have been typically used for forest restoration in the hilly area of southern China and accounted for considerable proportions of plantation forests in this region^35^.

Supplementary Table S1 lists major vegetation and soil properties of the four forests under study. In comparisons with the two plantations, the BF and CF had higher stem density, diameter at breast height (DBH), litterfall, and root biomass (Supplementary Table S1). In terms of coverage, tree height and aboveground net primary production (ANPP), the 25-year-old broadleaved species mixed plantation (BP) was close to the older primary and secondary forests (i.e., the BF and CF). Soils at the DBR sites had higher clay content, dissolved inorganic nitrogen (DIN) but lower available phosphorous (P) content (Supplementary Table S1).

### Field measurements

Three 10 × 10 m^2^ quadrats were established in each of the four forests. Each quadrat contained three 1 × 1 m^2^ plots, which were trenched for measuring R_h_. Trenching was conducted as follow. Briefly, four sides of each trench plot were dug and double-layer nylon net (100 meshes) used to cover the outside edges of trench plots. Then soil was refilled to the trenches accordingly. The depth of trenching was 50 cm in the BF and CF unless base rock was reached, and 50–70 cm in the CP and BP. A previous study observed that the most roots (<5 mm) distributed in the soils of 0–40 cm depth in forests in the study site, and therefore this depth was considered as main lateral root zone in these forests^61^. Moreover, some of trenches were down to base rock when established, especially in the CF. We therefore considered that in the present study, trenches were deep enough to exclude the most of lateral root respiration which accounted for the largest proportion of total root respiration^62^. In spite of lateral diffusion of root respired CO_2_ into trenches likely resulting in overestimation on R_h_^63^, nylon net was used to prevent roots from growing into those trenched plots while minimizing interruption on laterally water and nutrient exchanges; soil conditions in such trenches were closer to nature than that in trenches isolated by PVC panels since the latter was more likely to change soil conditions, e.g., water content in trenched plots and nutrient exchanges between trenched and their neighboring areas. In each plot, one 20 × 5 cm PVC collar (diameter × height) was inserted into soil, with the upper 2 cm sticking out above soil surface, and left *in situ* throughout the whole measurement period. Trenching and collar installations were conducted in November 2009. Small plants in these trenched plots were removed frequently in order to exclude R_a_.

Soil respiration rate was measured in each plot using a portable CO_2_ analyzer (Li-8100 Auto Soil CO_2_ Flux System, Li-Cor Biosciences, NE, US) for two years from February 2010 to January 2012, with a frequency of twice per month (normally on rainless days at the start and in the middle of a month). With auxiliary temperature and moisture sensors, soil temperature and moisture at 5 cm depth were recorded at the same time. At each sampling time, soil respiration rate was measured between 9:00 and 12:00 a.m. local time, during which the measured respiration rate was approximate to the daily averages in the study site^19^,^34^.

### Soil collections and analyses of soil properties

A circular soil auger (4 × 20 cm^2^; inner diameter × length) was used to collect soil samples in the trenched plots monthly at the second measuring time for R_s_ each month. For each sampling occasion, samples from the same quadrat were mixed homogenously to form a composite sample for lab analyses. Thus, we finally had three soil samples per stand each time.

Prior to soil assays, visible stones and roots were picked out and samples were sieved through a 2-mm mesh. Each sample was then separated into two parts, in which the first was used to determine DOC and MBC and the second air-dried and further ground to test ROC, TOC, and TN. In brief, MBC was assayed by fumigation-extraction-back titration method and calculated with an extraction coefficient of 0.38, following Vance *et al*.^64^. The K_2_SO_4_-extracted OC concentration in non-fumigated soils indicated DOC, which was a proxy of soil substrate availability^51^. ROC was determined using a wet oxidizing method with 0.333 *M* KMnO_4_, as described in Blair *et al*.^55^. TOC was analyzed with the Walkley-Black method and TN with the micro-Kjeldahl method^65^. Difference between TOC and ROC in each sample was content of NROC.

### Statistical analyses

Heterotrophic soil respiration rate in the same quadrat was averaged at each sampling occasion (*n* = 3) in prior to further analyses such as correlations and analysis of variance (ANOVA). To explore underlying influences of substrate quality on the R_h_ variations across forests, specific respiration rate was calculated via dividing respiration rate by content of organic carbon (OC)^23^, i.e., μmol CO_2_-C release per unit of OC, which reflected decomposability or quality of OC^36^,^37^, as well as microbial decomposition efficiency in soils^23^. The ratio of R_h_ over the corresponding DOC, ROC or TOC content was calculated in this study to indicate decomposability of OC^23^,^36^,^37^,^66^. This normalization process could cancel substrate quantity-induced differences in R_h_ and shed lights on effects of substrate quality on R_h_^23^,^36^,^37^. Microbial respiration quotient, i.e., the R_h_ per unit of MBC, was calculated to indicate metabolic status of soil microbes^66^,^67^. Repeated measures ANOVA were used to detect differences in respiration rate and soil physiochemical properties among forests over time. One-way ANOVA was also performed to explore significant difference of average R_h_ and edaphic properties across forests. Tukey HSD multiple comparisons were employed when difference was significant. Bivariate correlation was used to test potential relationships between R_h_ and soil properties. Taking into account of predominant effect of temperature on R_h_ over other environmental and soil physiochemical and microbial properties, partial correlation and partial mantel analyses were also employed to statistically check the relationships between R_h_ and soil properties when excluding their confounding effects with temperature. Data were tested for normality and heterogeneity of variance in prior to ANOVAs and correlations. Logarithm- or rank-transformation was performed once H_0_ assumption was rejected. For all the statistics, significance level was set at *P* < 0.05. All the statistical analyses, except for partial mantel tests conducted in R software (version 3.1.2), were finished in SPSS 16.0 for windows (SPSS Inc., Chicago, US) and graphs made in SigmaPlot 10.0 (Systat Software Inc., California, US).

## Additional Information

**How to cite this article**: Wei, H. *et al*. Are variations in heterotrophic soil respiration related to changes in substrate availability and microbial biomass carbon in the subtropical forests? *Sci. Rep*. **5**, 18370; doi: 10.1038/srep18370 (2015).

## Supplementary Material

Supplementary Information

## Figures and Tables

**Table 1:** Results of repeated measures ANOVA on heterotrophic soil respiration (R_h_) and the according soil properties across the forests over time.

		**Forest**	**Time**	**Forest & Time**
R_h_	*F*	10.26	21.46	1.53
	*P*	0.004	<0.001	0.162
T_5_	*F*	79.36	9870.7	92.3
	*P*	<0.001	<0.001	<0.001
M_5_	*F*	0.48	56.93	4.81
	*P*	0.708	<0.001	<0.001
MBC	*F*	66.64	1.6	2.42
	*P*	<0.001	0.204	0.028
DOC	*F*	8.26	24.59	2.01
	*P*	0.008	<0.001	0.043
ROC	*F*	36.98	5.07	1.59
	*P*	<0.001	0.001	0.12
TOC	*F*	50.4	8.41	2.18
	*P*	<0.001	<0.001	0.020
TN	*F*	93.71	10.42	1.83
	*P*	<0.001	<0.001	0.064

Numbers represent statistical *F* and *P* values, with bold indicating significant differences at *P* < 0.05. In the table, T_5_ and M_5_ are soil temperature and moisture at 5 cm depth, MBC microbial biomass carbon (C), DOC dissolved organic C, ROC readily-oxidizable organic C, TOC total organic C, and TN total nitrogen content.

## References

[b1] Raich J. W. & Schlesinger W. H. The global carbon dioxide flux in soil respiration and its relationship to vegetation and climate. Tellus B 44, 81–99, doi: 10.1034/j.1600-0889.1992.t01-1-00001.x (1992).

[b2] Raich J. W. & Potter C. S. Global patterns of carbon dioxide emissions from soils. Global Biogeochem. Cy. 9, 23–36, doi: 10.1029/94GB02723 (1995).

[b3] Jenkinson D. S., Adams D. E. & Wild A. Model estimates of CO_2_ emissions from soil in response to global warming. Nature 351, 304–306, doi: 10.1038/351304a0 (1991).

[b4] Luo Y. Terrestrial carbon-cycle feedback to climate warming. Annu. Rev. Ecol. Evol. S. 38, 683–712, doi: 10.1146/annurev.ecolsys.38.091206.095808 (2007).

[b5] Bond-Lamberty B. & Thomson A. A global database of soil respiration data. Biogeosciences 7, 1915–1926, doi: 10.5194/bg-7-1915-2010 (2010).

[b6] Hanson P., Edwards N., Garten C. & Andrews J. Separating root and soil microbial contributions to soil respiration: a review of methods and observations. Biogeochemistry 48, 115–146, doi: 10.1023/A:1006244819642 (2000).

[b7] Ryan M. G. & Law B. E. Interpreting, measuring, and modeling soil respiration. Biogeochemistry 73, 3–27, doi: 10.1007/s10533-004-5167-7 (2005).

[b8] Moyano F. E., Manzoni S. & Chenu C. Responses of soil heterotrophic respiration to moisture availability: An exploration of processes and models. Soil Biol. Biochem. 59, 72–85, doi: 10.1016/j.soilbio.2013.01.002 (2013).

[b9] Davidson E. A., Verchot L. V., Cattanio J. H., Ackerman I. L. & Carvalho J. E. M. Effects of soil water content on soil respiration in forests and cattle pastures of eastern Amazonia. Biogeochemistry 48, 53–69, doi: 10.1023/A:1006204113917 (2000).

[b10] Yan J., Zhang D., Zhou G. & Liu J. Soil respiration associated with forest succession in subtropical forests in Dinghushan Biosphere Reserve. Soil Biol. Biochem. 41, 991–999, doi: 10.1016/j.soilbio.2008.12.018 (2009).

[b11] Wang W. J., Dalal R. C., Moody P. W. & Smith C. J. Relationships of soil respiration to microbial biomass, substrate availability and clay content. Soil Biol. Biochem. 35, 273–284, doi: 10.1016/S0038-0717(02)00274-2 (2003).

[b12] Janssens I. A. . Productivity overshadows temperature in determining soil and ecosystem respiration across European forests. Global Change Biol. 7, 269–278, doi: 10.1046/j.1365-2486.2001.00412.x (2001).

[b13] Ekberg A., Buchmann N. & Gleixner G. Rhizospheric influence on soil respiration and decomposition in a temperate Norway spruce stand. Soil Biol. Biochem. 39, 2103–2110, doi: 10.1016/j.soilbio.2007.03.024 (2007).

[b14] Wild B. . Input of easily available organic C and N stimulates microbial decomposition of soil organic matter in arctic permafrost soil. Soil Biol. Biochem. 75, 143–151, doi: 10.1016/j.soilbio.2014.04.014 (2014). 25089062 PMC4064687

[b15] De Nobili M., Contin M., Mondini C. & Brookes P. C. Soil microbial biomass is triggered into activity by trace amounts of substrate. Soil Biol. Biochem. 33, 1163–1170, doi: 10.1016/S0038-0717(01)00020-7 (2001).

[b16] Thiessen S., Gleixner G., Wutzler T. & Reichstein M. Both priming and temperature sensitivity of soil organic matter decomposition depend on microbial biomass – An incubation study. Soil Biol. Biochem. 57, 739–748, doi: 10.1016/j.soilbio.2012.10.029 (2013).

[b17] de Graaff M. A., Classen A. T., Castro H. F. & Schadt C. W. Labile soil carbon inputs mediate the soil microbial community composition and plant residue decomposition rates. New Phytol. 188, 1055–1064, doi: 10.1111/j.1469-8137.2010.03427.x (2010). 21058948

[b18] Wan S. & Luo Y. Substrate regulation of soil respiration in a tallgrass prairie: Results of a clipping and shading experiment. Global Biogeochem. Cy. 17, 1054, doi: 10.1029/2002gb001971 (2003).

[b19] Yan J., Wang Y., Zhou G. & Zhang D. Estimates of soil respiration and net primary production of three forests at different succession stages in South China. Global Change Biol. 12, 810–821, doi: 10.1111/j.1365-2486.2006.01141.x (2006).

[b20] Peterson F. S. & Lajtha K. J. Linking aboveground net primary productivity to soil carbon and dissolved organic carbon in complex terrain. Journal of Geophysical Research: Biogeosciences 18, 1225–1236 doi: 10.1002/jgrg.20097 (2013).

[b21] Iqbal J. . Microbial biomass, and dissolved organic carbon and nitrogen strongly affect soil respiration in different land uses: A case study at Three Gorges Reservoir Area, South China. Agr. Ecosyst. Environ. 137, 294–307, doi: 10.1016/j.agee.2010.02.015 (2010).

[b22] He Y. . Uncertainty in the fate of soil organic carbon: A comparison of three conceptually different decomposition models at a larch plantation. Journal of Geophysical Research: Biogeosciences 119, 1892–1905, doi: 10.1002/2014jg002701 (2014).

[b23] Fang C. & Moncrieff J. B. The variation of soil microbial respiration with depth in relation to soil carbon composition. Plant Soil 268, 243–253, doi: 10.1007/s11104-004-0278-4 (2005).

[b24] Cheng W. X., Zhang Q. L., Coleman D. C., Carroll C. R. & Hoffman C. A. Is available carbon limiting microbial respiration in the rhizosphere? Soil Biol. Biochem. 28, 1283–1288, doi: 10.1016/S0038-0717(96)00138-1 (1996).

[b25] Lawrence C. R., Neff J. C. & Schimel J. P. Does adding microbial mechanisms of decomposition improve soil organic matter models? A comparison of four models using data from a pulsed rewetting experiment. Soil Biol. Biochem. 41, 1923–1934, doi: 10.1016/j.soilbio.2009.06.016 (2009).

[b26] Luo Y., Keenan T. F. & Smith M. Predictability of the terrestrial carbon cycle. Global Change Biol. 21, 1737–1751, doi: 10.1111/gcb.12766 (2014). 25327167

[b27] Wieder W. R., Bonan G. B. & Allison S. D. Global soil carbon projections are improved by modelling microbial processes. Nature Climate Change 3, 909–912, doi: 10.1038/nclimate1951 (2013).

[b28] Davidson E. A., Samanta S., Caramori S. S. & Savage K. The Dual Arrhenius and Michaelis-Menten kinetics model for decomposition of soil organic matter at hourly to seasonal time scales. Global Change Biol. 18, 371–384, doi: 10.1111/j.1365-2486.2011.02546.x (2012).

[b29] Fan Z. & Liang C. Significance of microbial asynchronous anabolism to soil carbon dynamics driven by litter inputs. Scientific Reports 5, 9575, doi: 10.1038/srep09575 (2015). 25849864 PMC4387914

[b30] Todd-Brown K. E. O., Hopkins F. M., Kivlin S. N., Talbot J. M. & Allison S. D. A framework for representing microbial decomposition in coupled climate models. Biogeochemistry 109, 19–33, doi: 10.1007/s10533-011-9635-6 (2012).

[b31] Cotrufo M. F., Wallenstein M. D., Boot C. M., Denef K. & Paul E. The Microbial Efficiency-Matrix Stabilization (MEMS) framework integrates plant litter decomposition with soil organic matter stabilization: do labile plant inputs form stable soil organic matter? Global Change Biol. 19, 988–995, doi: 10.1111/gcb.12113 (2013). 23504877

[b32] Birge H. E. . Soil respiration is not limited by reductions in microbial biomass during long-term soil incubations. Soil Biol. Biochem. 81, 304–310, doi: 10.1016/j.soilbio.2014.11.028 (2015).

[b33] Piao S. . The carbon balance of terrestrial ecosystems in China. Nature 458, 1009–1013, doi: 10.1038/nature07944 (2009). 19396142

[b34] Tang X., Liu S., Zhou G., Zhang D. & Zhou C. Soil-atmospheric exchange of CO_2_, CH_4_, and N_2_O in three subtropical forest ecosystems in southern China. Global Change Biol. 12, 546–560, doi: 10.1111/j.1365-2486.2006.01109.x (2006).

[b35] Liu H. . Greenhouse gas fluxes from soils of different land-use types in a hilly area of South China. Agr. Ecosyst. Environ. 124, 125–135, doi: 10.1016/j.agee.2007.09.002 (2008).

[b36] Lee H., Schuur E. A. G., Inglett K. S., Lavoie M. & Chanton J. P. The rate of permafrost carbon release under aerobic and anaerobic conditions and its potential effects on climate. Global Change Biol. 18, 515–527, doi: 10.1111/j.1365-2486.2011.02519.x (2012).

[b37] Treat C. C. . A pan-Arctic synthesis of CH_4_ and CO_2_ production from anoxic soil incubations. Global Change Biol. 21, 2787–2803, doi: 10.1111/gcb.12875 (2015). 25620695

[b38] Yi Z. . Partitioning soil respiration of subtropical forests with different successional stages in south China. Forest Ecol. Manag. 243, 178–186, doi: 10.1016/j.foreco.2007.02.022 (2007).

[b39] Corlett R. T. Where are the Subtropics? Biotropica 45, 273–275, doi: 10.1111/btp.12028 (2013).

[b40] Schulze E. D. Soil respiration of tropical vegetation types. Ecology 48, 652–653, doi: 10.2307/1936509 (1967).

[b41] Curiel Yuste J., Janssens I. A., Carrara A. & Ceulemans R. Annual Q_10_ of soil respiration reflects plant phenological patterns as well as temperature sensitivity. Global Change Biol. 10, 161–169, doi: 10.1111/j.1529-8817.2003.00727.x (2004).

[b42] Davidson E. A., Janssens I. A. & Luo Y. Q. On the variability of respiration in terrestrial ecosystems: moving beyond Q_10_. Global Change Biol. 12, 154–164, doi: 10.1111/j.1365-2486.2005.01065.x (2006).

[b43] Wang C. K., Yang J. Y. & Zhang Q. Z. Soil respiration in six temperate forests in China. Global Change Biol. 12, 2103–2114, doi: 10.1111/j.1365-2486.2006.01234.x (2006).

[b44] Vicca S. . Can current moisture responses predict soil CO_2_ efflux under altered precipitation regimes? A synthesis of manipulation experiments. Biogeosciences 11, 2991–3013, doi: 10.5194/bg-11-2991-2014 (2014).

[b45] Jiang H. . Responses of soil respiration and its temperature/moisture sensitivity to precipitation in three subtropical forests in southern China. Biogeosciences 10, 3963–3982, doi: 10.5194/bg-10-3963-2013 (2013).

[b46] Deng Q. . Responses of soil respiration to elevated carbon dioxide and nitrogen addition in young subtropical forest ecosystems in China. Biogeosciences 7, 315–328, doi: 10.5194/bg-7-315-2010 (2010).

[b47] Campbell J. L. & Law B. E. Forest soil respiration across three climatically distinct chronosequences in Oregon. Biogeochemistry 73, 109–125, doi: 10.1007/s10533-004-5165-9 (2005).

[b48] Tedeschi V. . Soil respiration in a Mediterranean oak forest at different developmental stages after coppicing. Global Change Biol. 12, 110–121, doi: 10.1111/j.1365-2486.2005.01081.x (2006).

[b49] Wei H., Xiao G. L., Guenet B., Janssens I. A. & Shen W. J. Soil microbial community composition does not predominantly determine the variance of heterotrophic soil respiration across four subtropical forests. Scientific Reports 5, 7854, doi: 10.1038/Srep07854 (2015). 25598010 PMC4297967

[b50] Cook B. D. & Allan D. L. Dissolved organic carbon in old field soils - total amounts as a measure of available resources for soil mineralization. Soil Biol. Biochem. 24, 585–594, doi: 10.1016/0038-0717(92)90084-B (1992).

[b51] Boyer J. N. & Groffman P. M. Bioavailability of water extractable organic carbon fractions in forest and agricultural soil profiles. Soil Biol. Biochem. 28, 783–790, doi: 10.1016/0038-0717(96)00015-6 (1996).

[b52] Neff J. C. & Asner G. P. Dissolved organic carbon in terrestrial ecosystems: synthesis and a model. Ecosystems 4, 29–48, doi: 10.1007/s100210000058 (2001).

[b53] Cleveland C. C., Neff J. C., Townsend A. R. & Hood E. Composition, dynamics, and fate of leached dissolved organic matter in terrestrial ecosystems: results from a decomposition experiment. Ecosystems 7, 275–285, doi: 10.1007/s10021-003-0236-7 (2004).

[b54] Bengtson P. & Bengtsson G. Rapid turnover of DOC in temperate forests accounts for increased CO_2_ production at elevated temperatures. Ecol. Lett. 10, 783–790, doi: 10.1111/j.1461-0248.2007.01072.x (2007). 17663711

[b55] Blair G. J., Lefrog R. D. B. & Lisle L. Soil carbon fractions based on their degree of oxidation, and the development of a carbon management index for agricultural systems. Aust. J. Agr. Res. 46, 1459–1466, doi: 10.1071/AR9951459 (1995).

[b56] Craine J. M., Morrow C. & Fierer N. Microbial nitrogen limitation increases decomposition. Ecology 88, 2105–2113, doi: 10.1890/06-1847.1 (2007). 17824441

[b57] Manzoni S., Taylor P., Richter A., Porporato A. & Ågren G. I. Environmental and stoichiometric controls on microbial carbon-use efficiency in soils. New Phytol. 196, 79–91, doi: 10.1111/j.1469-8137.2012.04225.x (2012). 22924405

[b58] Zhou G. . Quantifying the hydrological responses to climate change in an intact forested small watershed in Southern China. Global Change Biol. 17, 3736–3746, doi: 10.1111/j.1365-2486.2011.02499.x (2011).

[b59] Wang J., Ren H., Yang L. & Duan W. Establishment and early growth of introduced indigenous tree species in typical plantations and shrubland in South China. Forest Ecol. Manag. 258, 1293–1300, doi: 10.1016/j.foreco.2009.06.022 (2009).

[b60] Li Z., Peng S., Rae D. J. & Zhou G. Litter decomposition and nitrogen mineralization of soils in subtropical plantation forests of southern China, with special attention to comparisons between legumes and non-legumes. Plant Soil 229, 105–116, doi: 10.1023/A:1004832013143 (2001).

[b61] Wen D., Wei P., Kong G. & Ye W. Production and turnover rate of fine roots in two lower subtropical forest sites at Dinghushan. Chinese Journal of Plant Ecology 23, 361–369 (1999).

[b62] Chen D. M., Zhou L. X., Rao X. Q., Lin Y. B. & Fu S. L. Effects of root diameter and root nitrogen concentration on *in situ* root respiration among different seasons and tree species. Ecol. Res. 25, 983–993, doi: 10.1007/s11284-010-0722-2 (2010).

[b63] Jassal R. S. & Black T. A. Estimating heterotrophic and autotrophic soil respiration using small-area trenched plot technique: Theory and practice. Agr. Forest Meteorol. 140, 193–202, doi: 10.1016/j.agrformet.2005.12.012 (2006).

[b64] Vance E. D., Brookes P. C. & Jenkinson D. An extraction method for measuring soil microbial biomass C. Soil Biol. Biochem. 19, 703–707, doi: 10.1016/0038-0717(87)90052-6 (1987).

[b65] Liu G. Soil physicochemical analysis and description of soil profiles. (China Normative Publishing House, 1996).

[b66] Wei H. . High clay content accelerates the decomposition of fresh organic matter in artificial soils. Soil Biol. Biochem. 77, 100–108, doi: 10.1016/j.soilbio.2014.06.006 (2014).

[b67] Anderson T.-H. & Domsch K. H. The metabolic quotient for CO_2_ (*q*CO_2_) as a specific activity parameter to assess the effects of environmental conditions, such as pH, on the microbial biomass of forest soils. Soil Biol. Biochem. 25, 393–395, doi: 10.1016/0038-0717(93)90140-7 (1993).

