# Supplementary material for: Are variations in heterotrophic soil respiration related to changes in substrate availability and microbial biomass carbon in the subtropical forests?
Source: Sci Rep. 2015 Dec 16;5:18370. doi: 10.1038/srep18370 (PMC4680953; doi:10.1038/srep18370)
Supplement: Supplementary Information [file srep18370-s1.pdf]

# Supplementary materials

Correspondence and requests for materials should be addressed to W.S. (shenweij@scbg.ac.cn)

# Are variations in heterotrophic soil respiration related to changes in substrate availability and microbial biomass carbon in the subtropical forests?

Hui Wei [1,†], Xiaomei Chen [2,†], Guoliang Xiao [1,5], Bertrand Guenet [3], Sara Vicca [4] , Weijun Shen [1,*]

[1] Key Laboratory of Vegetation Restoration and Management of Degraded Ecosystems, South China Botanical Garden, Chinese Academy of Sciences, Guangzhou 510650, China

[2] School of Geographical Sciences, Guangzhou University, Guangzhou 510006, China

[3] Laboratoire des Sciences du Climat et de l'Environnement, Centre National de Recherche Scientifique, Gif Sur Yvette 91190, France

[4] Research Group of Plant and Vegetation Ecology, Department of Biology, University of Antwerp, Universiteitsplein 1, 2610 Wilrijk, Belgium

[5] University of Chinese Academy of Sciences, Beijing 100049, China

[†] These authors contributed equally to this study.

[*] Corresponding author: Dr. Weijun Shen

Key Laboratory of Vegetation Restoration and Management of Degraded Ecosystems,

South China Botanical Garden, Chinese Academy of Sciences

723 Xingke Road, Tianhe District

Guangzhou 510650,

China

Tel.: + 86 20 3725 2950;

Fax: + 86 20 3725 2950;

Email: shenweij@scbg.ac.cn

**Supplementary Table S1| Major vegetation and soil characteristics of the four forests.** DBH: tree diameter at breast height; ANPP: above-ground net primary production; DIN: dissolved inorganic nitrogen. BF represents the primary evergreen broadleaved forest, CF the secondary forest mixed with coniferous and broadleaved tree species, CP the plantation mixed with coniferous tree species, and BP the plantation mixed with native broadleaved tree species. [*] From Fang et al. (2009); [•] From Fang et al. (2005); [ǂ] From Yan et al. (2006); [†] From Huang et al. (2011); [‡] From Zhou et al. (2005); [ǀ] From Tang et al. (2006); [¶] From Shen et al. (2001) and Li et al. (2001); [§] From Fu et al. (2011).

| Forest type | BF | CF | CP | BP |
|---|---|---|---|---|
| Stand age (years) | > 400 | ~ 100 | 25 | 25 |
| Coverage (%) | > 95% [*] | > 90% [*] | 70% [¶] | ~ 90% [¶] |
| Stem density (tree $ha^{-1}$) | 1729 [•] | 1933 [•] | 1330 [¶] | 1600 [¶] |
| Mean tree height (m) | 10.0 [*] | 7.7 [*] | 7.2 [¶] | 9.5 [§] |
| DBH (cm) | 18.5 [*] | 14.2 [*] | 12.6 [¶] | 14.5 [§] |
| ANPP (g C $m^{-2}$ $yr^{-1}$) | 438 [ǂ] | 424 [ǂ] | 173 [§] | 458 [§] |
| Litterfall (kg $m^{-2}$ $yr^{-1}$) | 0.8 [†] | 0.9 [†] | 0.3 [§] | 0.6 [§] |
| Root biomass (kg $m^{-2}$) | 9.6 [‡] | 8.8 [‡] | 2.5 [§] | 3.1 [§] |
| Bulk density (g $cm^{-3}$) | 1.1 [ǀ] | 1.2 [ǀ] | 1.4 [§] | 1.4 [§] |
| Clay (wt. %) | 40.5 [*] | 33.8 [*] | 21.7 [§] | 16.1 [§] |
| Silt (wt. %) | 34.7 [*] | 29.4 [*] | 19.3 [§] | 22.1 [§] |
| Sand (wt. %) | 24.8 [*] | 36.8 [*] | 46.6 [§] | 47.3 [§] |
| DIN (mg $kg^{-1}$) | 17.4 [ǀ] | 18.6 [ǀ] | 6.6 [§] | 7.3 [§] |
| Available P (mg $kg^{-1}$) | 1.8 [†] | 1.1 [†] | 2.1 [§] | 1.8 [§] |

**Supplementary Table S2 | Comparison of annual heterotrophic soil respiration ($R_h$) and its temperature sensitivity ($Q_{10}$) across Chinese subtropical forests.** The unit of $R_h$ is Mg C $ha^{-1}$ $yr^{-1}$.

| Forest type | Annual $R_h$ | $Q_{10}$ | Literature |
|---|---|---|---|
| Monsoon evergreen broadleaved forest | 5.9 | 1.9 | This study |
| Natural evergreen broadleaved forest | 7.3 | | Yang et al., 2007 |
| Monsoon evergreen broadleaved forest | 4.9 | <1.5 | Yi et al., 2007 |
| Natural evergreen broadleaved forest | 8.2 | 2.7 | Tan et al., 2013 |
| Mixed conifer and broadleaf forest | 5.7 | 1.7 | This study |
| Mixed conifer and broadleaf forest | 4.5 | <1.5 | Yi et al., 2007 |
| Mixed conifer plantation | 4.3 | 2.0 | This study |
| Chinese fir plantation | 2.8 | | Yang et al., 2007 |
| Pine forest | 4.3 | <1.5 | Yi et al., 2007 |
| Evergreen broadleaved plantation | 4.9 | 1.7 | This study |
| Average in global subtropical forest | 4.1 | | Bond-Lamberty & Thomson, 2010 |

## SUPPLEMENTARY FIGURE CAPTIONS

**Supplementary Fig. S1 | Daily precipitation and air temperature at 2 m height range from January 2010 to February 2012**. Data are originated from the Gaoyao weather station and downloaded in the website 'http://rp5.ru/Weather_archive_in_Gaoyao'. The Gaoyao weather station is around 20 km away from the Dinghushan site and 50 km away from the Heshan site in this study.

**Supplementary Fig. S2 | Bi-plots between soil temperature ($T_5$) measured at the studied forests and air temperature ($T_{air}$) recorded in the Gaoyao weather station**. Solid lines indicate linear relationships between the two variables in each forest. Statistical *r* is Spearman' correlation coefficients and *P* is significant level.

**Supplementary Fig. S3 | Seasonal patterns of microbial biomass carbon (MBC), readily-oxidizable organic carbon (ROC), non-readily oxidizable organic carbon (NROC), total organic carbon (TOC), and total nitrogen (TN) in the four forests throughout the measure period**. The BF is the monsoon evergreen broadleaved forest, CF the mixed coniferous and broadleaved forest, CP the plantation with mixed coniferous tree species, and BP the plantation with mixed native broadleaved tree species. The abbreviations for the four forests (BF, CF, CP, and BP) are the same in the following figures.

**Supplementary Fig. S4 | Bi-plots between heterotrophic soil respiration ($R_h$) and soil substrate content or microbial biomass carbon (MBC) in the four forests.** ROC is readily-oxidizable organic carbon (C), TOC total organic C, and TN total nitrogen.

**Supplementary Fig. S5 | Bi-plots between heterotrophic soil respiration ($R_h$) and soil substrate content or ratio of carbon over nitrogen (C/N) in the cool or warm season across the four forests.** Plots stand for means and error bars for standard errors (n=6). The warm season ranges from April to September while the other months in a year constitute the cool season. DOC is dissolved organic carbon (C), ROC readily-oxidizable organic C, TOC total organic carbon, and TN total nitrogen.

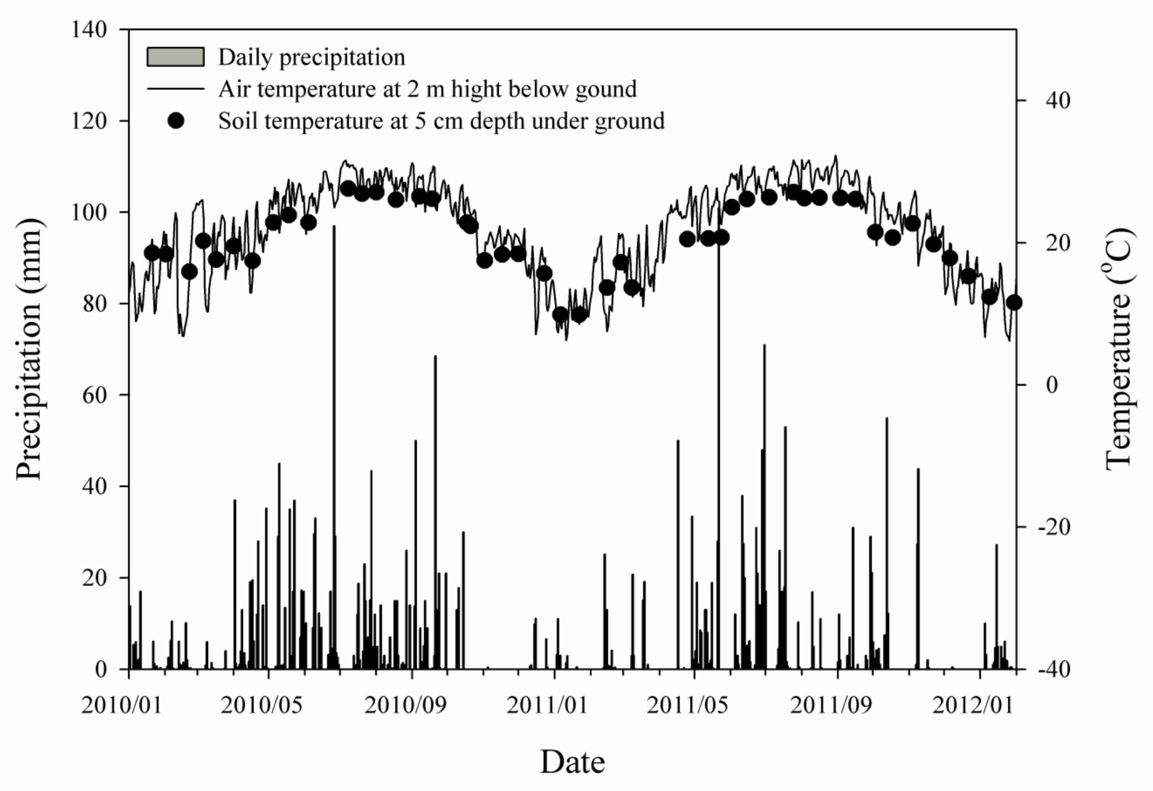


**Supplementary Fig. S1**

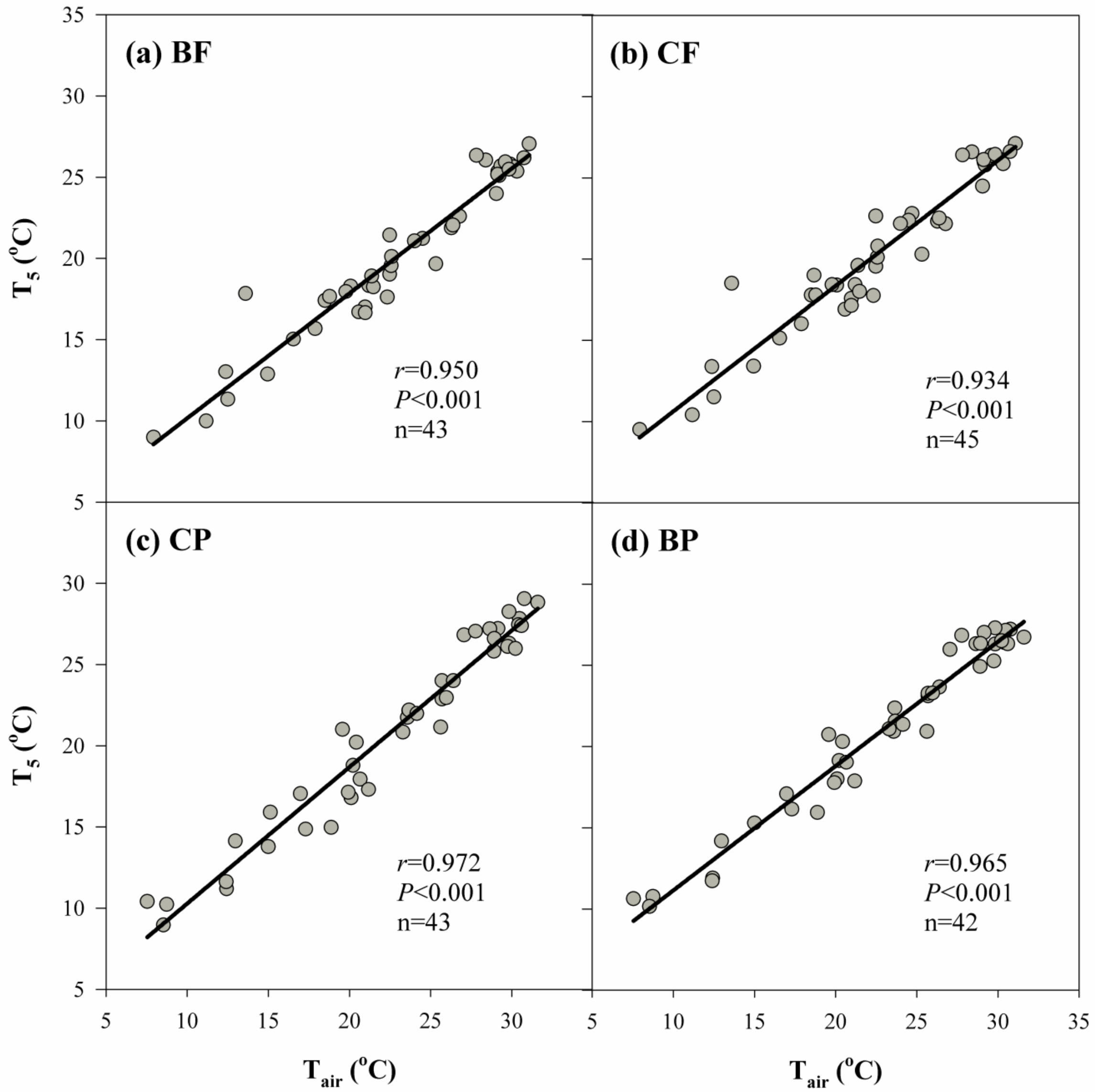


**Supplementary Fig S2**

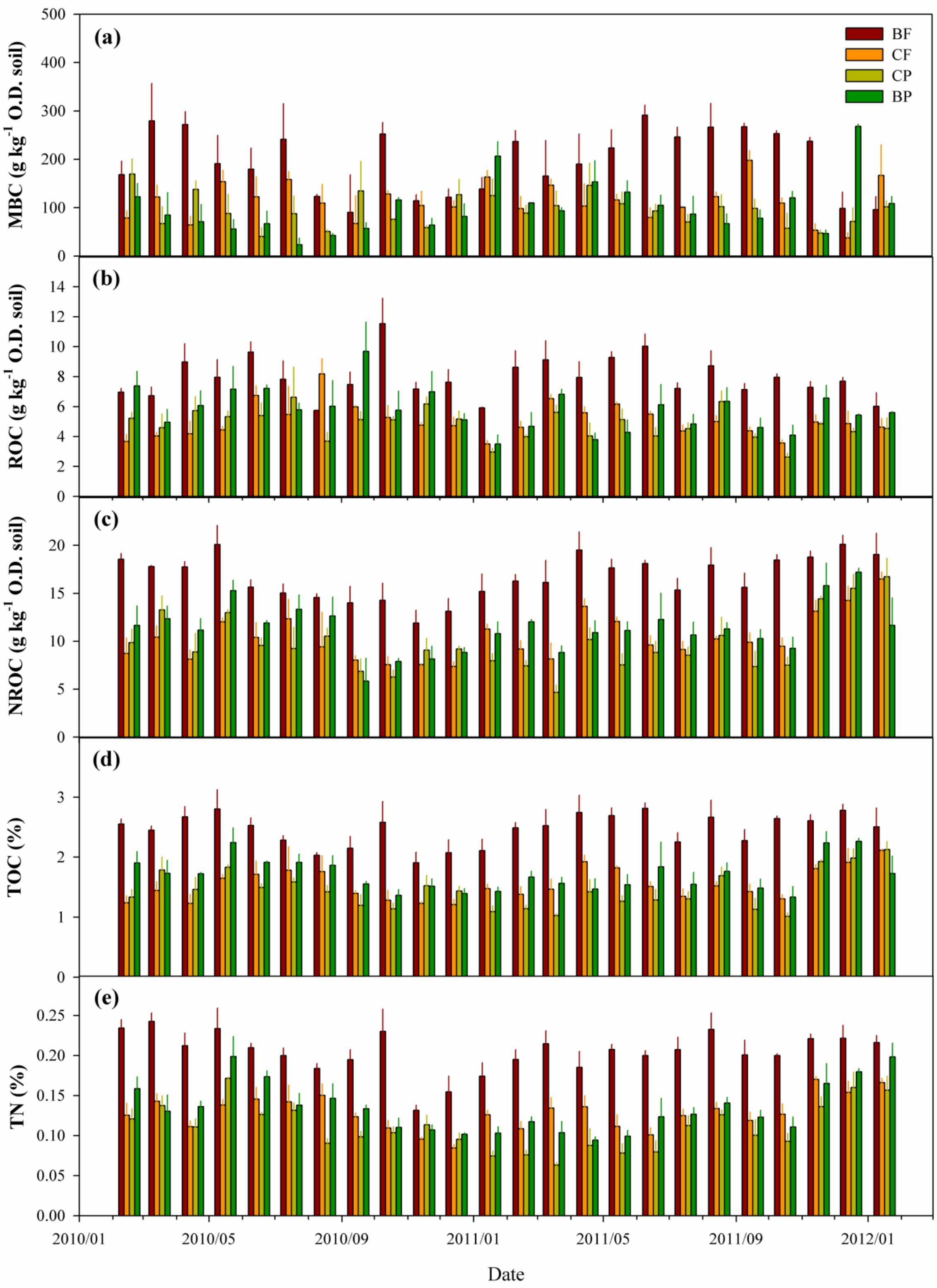


**Supplementary Fig. S3**

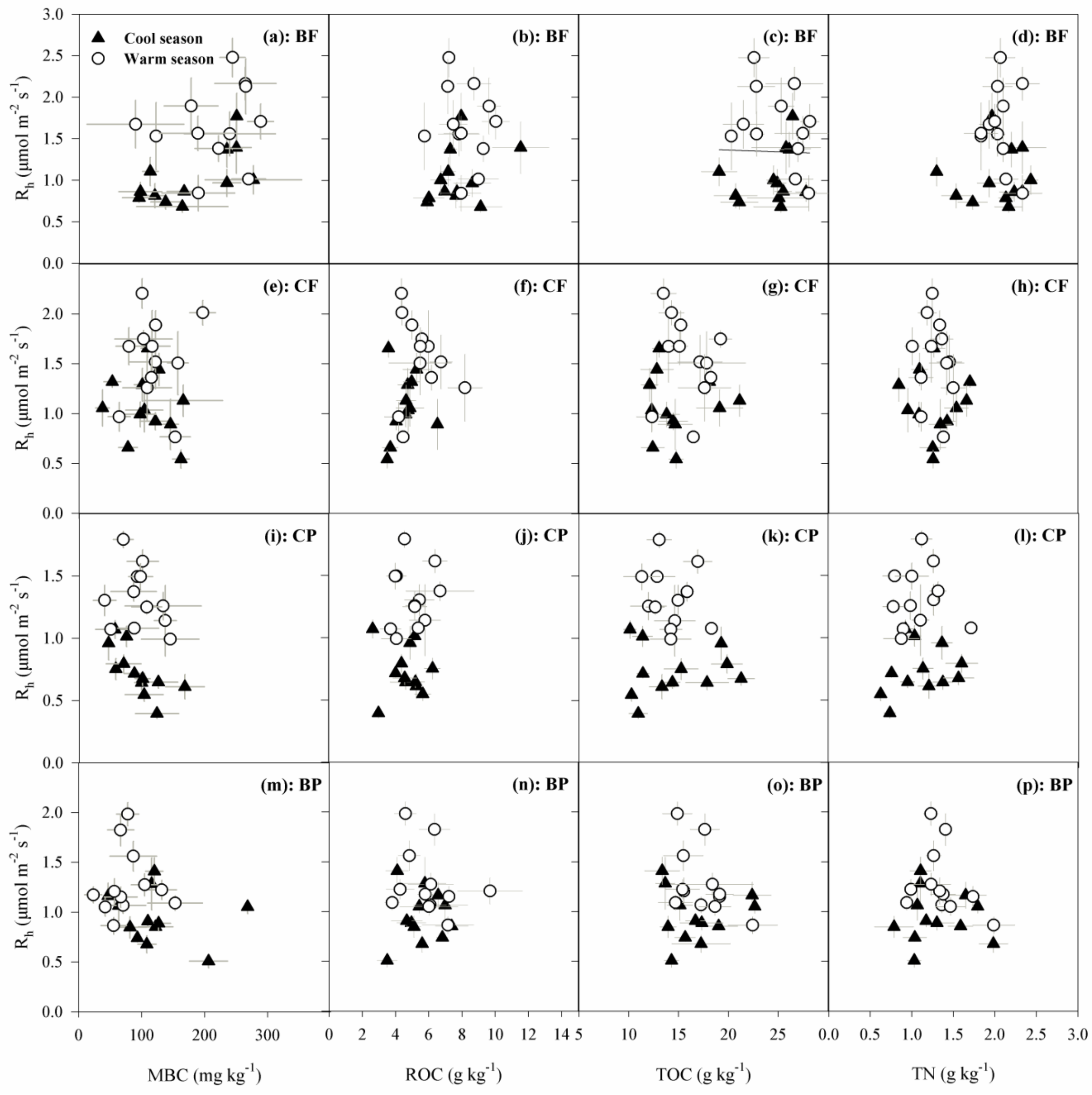


**Supplementary Fig S4**

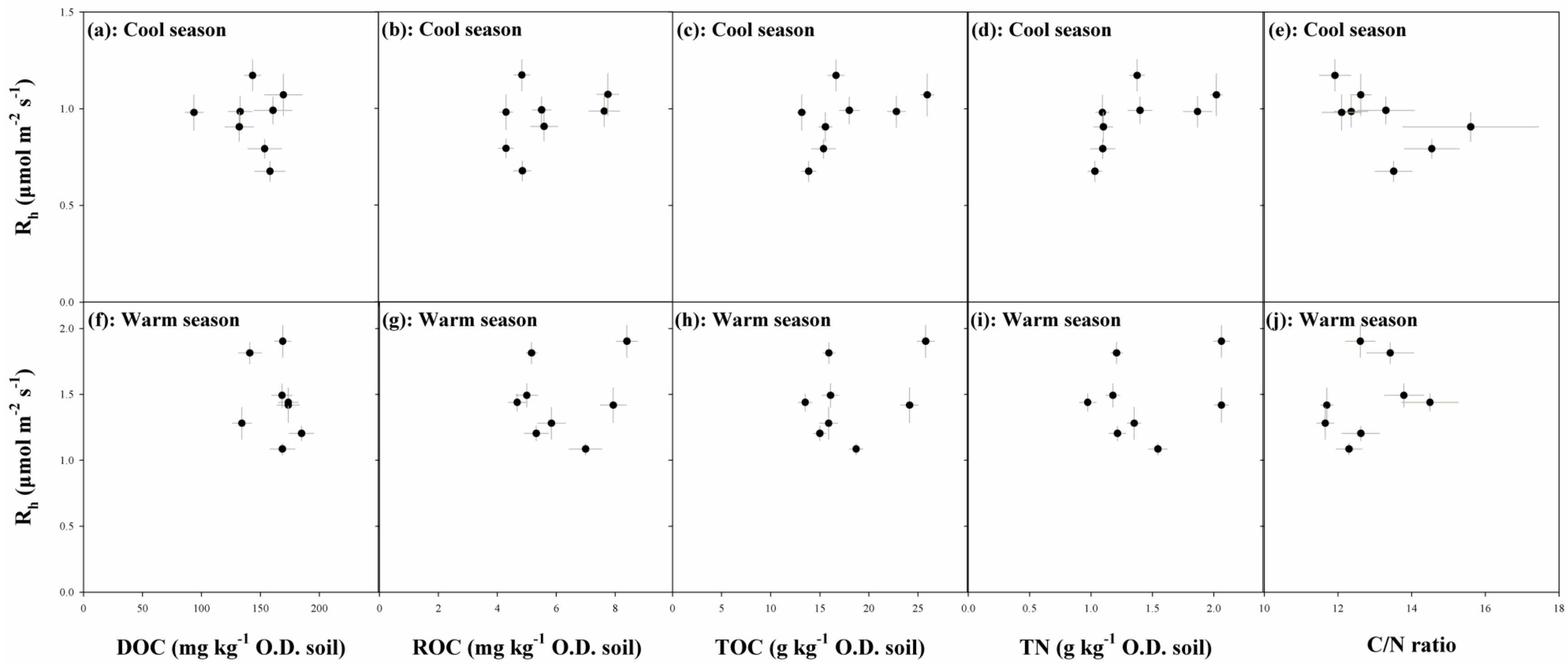


**Supplementary Fig S5**

## Reference

1 Fu, S., Lin, Y., Rao, X. & Liu, S. *Investigation and research dataset of stand properties in Chinese forest ecosystems - Heshan National Field Reseach station of Forest Ecosystem (1998 - 2008)*. (China Agriculture Press, 2011).

2 Fang, Y., Mo, J., Zhou, G. & Xue, J. Response of diameter at breast height increment to N additions in forests of Dinghushan Biosphere Reserve. *Journal of Tropical and Subtropical Botany* **13**, 198-204 (2005).

3 Li, Z., Peng, S., Rae, D. J. & Zhou, G. Litter decomposition and nitrogen mineralization of soils in subtropical plantation forests of southern China, with special attention to comparisons between legumes and non-legumes. *Plant Soil* **229** (2001).

4 Shen, W., Peng, S., Zhou, G., Lin, Y. & Li, Z. Ecohydrological functions of litter in man-made *Acacia mangium* and *Pinus elliotii* plantations. *Acta Ecologica Sinica* **5**, 846-850 (2001).

5 Fang, H. *et al.* $^{13}C$ abundance, water-soluble and microbial biomass carbon as potential indicators of soil organic carbon dynamics in subtropical forests at different successional stages and subject to different nitrogen loads. *Plant Soil* **320**, 243-254 (2009).

6 Huang, W., Liu, J., Zhou, G., Zhang, D. & Deng, Q. Effects of precipitation on soil acid phosphatase activity in three successional forests in Southern China. *Biogeosciences* **8**, 1901-1910 (2011).

7 Zhou, C., zhou, G., Zhang, D., Wang, Y. & Liu, S. $CO_2$ efflux from different forest soils and impact factors in Dinghu Mountain, China. *Sci China Ser D* **48**, 198-206 (2005).

8 Yan, J., Wang, Y., Zhou, G. & Zhang, D. Estimates of soil respiration and net primary production of three forests at different succession stages in South China. *Global Change Biol* **12**, 810-821 (2006).

9 Tang, X., Liu, S., Zhou, G., Zhang, D. & Zhou, C. Soil-atmospheric exchange of $CO_2$, $CH_4$, and $N_2O$ in three subtropical forest ecosystems in southern China. *Global Change Biol* **12**, 546-560 (2006).

10 Yang, Y. S., Chen, G. S., Guo, J. F., Xie, J. S. & Wang, X. G. Soil respiration and carbon balance in a subtropical native forest and two managed plantations. *Plant Ecol* **193**, 71-84 (2007).

11 Yi, Z. *et al.* Partitioning soil respiration of subtropical forests with different successional stages in south China. *Forest Ecol Manag* **243**, 178-186 (2007).

12 Tan, Z. *et al.* Soil respiration in an old-growth subtropical forest: Patterns, components, and controls. *Journal of Geophysical Research: Atmospheres* **118**, 2981-2990 (2013).

13 Bond-Lamberty, B. & Thomson, A. A global database of soil respiration data. *Biogeosciences* **7**, 1915-1926 (2010).
